# Use of the T-1470 LiteTouch™ Laser in the En Bloc Resection of an Upper Tract Urothelial Cancer

**DOI:** 10.1155/2021/6623326

**Published:** 2021-01-26

**Authors:** Ezra Shoen, Benjamin Zollinger, Tripp Gresham, Katayoon M. Rezaei, Michael Whalen

**Affiliations:** School of Medicine and Health Sciences, The George Washington University, Washington, D.C., 20037, USA

## Abstract

**Background:**

Endoscopic laser-ablative therapy of upper tract urothelial carcinoma offers kidney-sparing treatment for well-selected low-risk tumors. The traditional technique consists of tumor biopsy with flexible forceps or nitinol basket for pathologic assessment of stage and grade, followed by laser ablation of the tumor. In this case, we present the use of the new T-1470 LiteTouch™ laser for intraoperative tumor en bloc resection, affording both tissue acquisition and tumor ablation. *Case Presentation*. An 81-year-old female with a past medical history significant for stage 4 chronic kidney disease, peripheral artery disease, coronary artery disease, type 2 diabetes mellitus, and gout was diagnosed with a 2 cm left upper tract high-grade papillary urothelial carcinoma confirmed by cytology with cell block preparation. Using a novel approach, the tumor was resected, en bloc, using the T-1470 LiteTouch™ laser which allowed for sufficient tissue resection for pathologic examination and strong hemostasis. This new technique is the first recorded example of tumor en bloc resection using the T-1470 LiteTouch™ laser of an upper tract urothelial carcinoma.

**Conclusion:**

The use of the T-1470 LiteTouch™ laser offers promise for its use as a novel laser for the endoscopic treatment of upper tract urothelial carcinoma. It shows potential for advantages over current techniques through its ability to achieve en bloc resection and superior hemostasis.

## 1. Introduction

Endoscopic management of an upper tract urothelial carcinoma (UTUC) allows for kidney preservation compared to the historical standard of care of a radical nephroureterectomy (RNU), and is therefore an attractive treatment option for patients with underlying chronic kidney disease. The standard technique for endoscopic UTUC surgery includes tumor biopsy by forceps or nitinol basket, and then tumor ablation by holmium:yttrium-aluminum-garnet (Ho:YAG) and/or neodymium:yttrium-aluminum-garnet (Nd:YAG) lasers [1, 2]. In this study, we present the use of a novel laser, the T-1470 LiteTouch™ diode laser, produced by Convergent Laser Technologies, for the en bloc resection of a UTUC.

## 2. Case Presentation

An 81-year-old female with stage 4 chronic kidney disease, peripheral artery disease, coronary artery disease, type 2 diabetes mellitus, and gout was referred for management of suspected left upper tract malignancy in the setting of gross hematuria. Noncontrast CT abdomen and pelvis demonstrated mild left hydronephrosis and high-attenuation tissue density material in the left upper pole calyx, concerning for blood products and/or malignancy.

**Figure 1:**
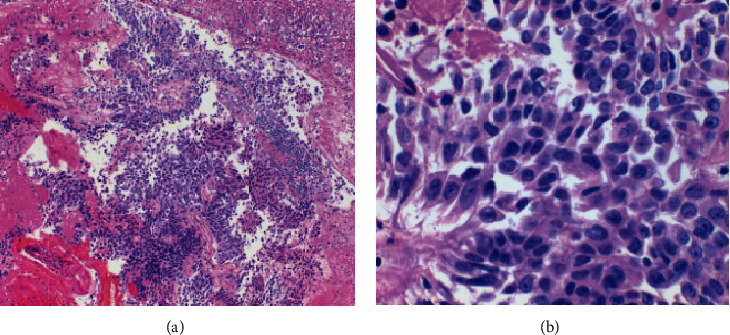
(a) Papillary urothelial carcinoma fragments; cell block (H&E, medium power 10x). (b) High-grade cytologic atypia with enlarged hyperchromatic nuclei and high N/C ratio; cell block (H&E, high power 40x).

After informed consent, the patient was taken to the operating room for cystoscopy, ureteroscopy, and biopsy. Intraoperatively, at least 2 cm left upper pole/renal pelvis tumor nodule was observed with surrounding blood clot, obscuring the true extent of the disease burden. Tissue was obtained with the Piranha forceps and brush biopsy. An attempt was made with the Ho:YAG laser to fulgurate the lesion, but total ablation was not performed given the obscuring clot burden. A ureteral stent was placed. Postoperatively, she did well. Cytologic examination with cell block preparation demonstrated high-grade urothelial carcinoma among nonviable papillary fragments (Figures 1(a) and 1(b)). Results from cytology/biopsy found no evidence of invasion and had a preliminary stage of pTa. This staging, however, may not have been fully representative of the tumor. A subsequent nuclear medicine renal scan revealed equal split renal function, increasing the risk of hemodialysis after standard of care nephroureterectomy. She therefore refused this treatment option, along with percutaneous resection and endocavitary therapy. After being lost to follow-up for several months, restaging cross-sectional imaging showed no retroperitoneal lymphadenopathy or metastatic disease. Again refusing percutaneous antegrade resection, she consented to ureteroscopy with laser ablation. The T-1470 LiteTouch™ laser was selected for performance characteristics, including superior efficacy of hemostasis compared to the Ho:YAG laser.

**Figure 2:**
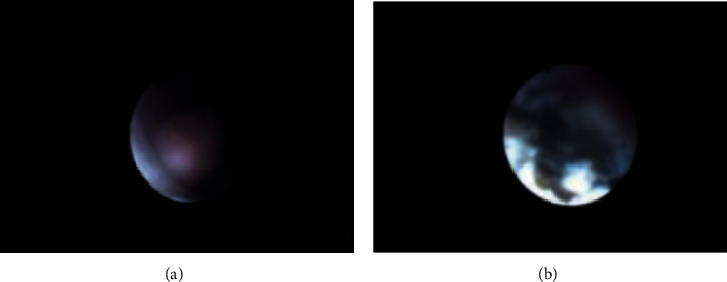
(a) Uteroscopic view of UTUC prior to en bloc resection. (b) Uteroscopic view of UTUC after en bloc resection. Hemostasis was achieved with no evidence of bleeding postresection.

The patient returned to the operating room for left ureteroscopy and biopsy with placement of an 11/13 ureteral access sheath and intended tumor laser ablation and ureteral stent placement. However, upon commencing the ablation, it was discovered that the laser was adept at creating a controlled and even tissue plane deep to the urothelium that enabled enucleation of the tumor largely en bloc (Figures 2(a) and 2(b)).

At the conclusion of the procedure, the enucleation bed was inspected and found to be hemostatic without concern of perforation (Figure 2(b)). Three large fragments of the tumor were evacuated with a nitinol basket via the ureteral access sheath and sent for pathological analysis. A ureteral stent was placed, and the case was concluded. Cytologic examination with cell block preparation again demonstrated high-grade papillary urothelial carcinoma (Figures 1(a) and 1(b)). Although sufficient tissue had been acquired for a thorough assessment of grade, pathologic staging was confounded by cautery artifacts caused by the laser (see Discussion Literature Review below for lessons learned). The patient is currently awaiting adjuvant endocavitary chemotherapy.

## 3. Discussion and Literature Review

To our knowledge, this case represents the first use of the T-1470 LiteTouch™ laser for UTUC treatment. While traditionally lasers have been used solely for endoscopic tumor ablation, the T-1470 LiteTouch™ laser's unique advantages make it an ideal candidate for both en bloc resection and hemostasis. Some limitations of endoscopic management include limited tissue yield afforded by traditional biopsy instruments, as well as tumor understaging due to inadequate assessment of the depth of penetration.

The T-1470 LiteTouch™ Laser system is a diode laser with a wavelength of 1470 nm, allowing for deep soft tissue penetration into fat, as well as absorption by water. Additionally, the wavelengths are well absorbed by oxyhemoglobin which allows for effective coagulation. These characteristics therefore offer superior soft tissue penetration for tissue acquisition without significant bleeding that would otherwise obscure the ablation zone. Such real-time hemostasis affords accurate assessment and adjustment of the resection plane, which are integral features to afford successful tumor en bloc resection. Furthermore, en bloc resection increases the pathologic yield and minimizes tissue loss during pathologic processing. The combined use of the 11/13 ureteral access sheath allows for rapid, real-time extraction of the resulting large tissue fragments.

The ability to obtain accurate grade-diagnostic biopsies prior to tumor ablation is a documented limitation to endoscopic UTUC treatment [3]. Because endoscopic management is typically only appropriate for low-risk UTUC, an accurate stage and grade are vital for informing the clinical decision on whether RNU is necessary [4]. The use of the T-1470 LiteTouch™ laser for en bloc resection allows for the pathological staging and grading of the fully excised tissue instead of a pre-op biopsy by forceps or basket. While Ho:YAG or Nd:YAG lasers have ablative capabilities, given the very limited depth of tissue penetration as well as limited hemostatic potential, tumor en bloc resection is not reliably performed with these technologies. At 5 W, the power settings for this case, the T-1470 LiteTouch™ achieves a depth of penetration of roughly 0.4 mm. Given the limited anatomical working space during ureteroscopy, tumor en bloc resection has been challenging and relatively inaccessible in the past. Additionally, achieving hemostasis is always a concern for endoscopic UTUC surgery, and failure to do so can pose serious risks to patient safety. The presence of intraoperative bleeding could reduce or eliminate the visualization of the surgical field during the procedure. Moreover, it may be challenging to identify and coagulate the source of the bleed. While the Ho:YAG laser's hemostatic ability is appropriate in the enucleation of the prostate [5], the upper tract offers a smaller working space, making a laser with improved hemostasis a more ideal option. The T-1470 LiteTouch™ Laser's size, precision, tissue absorptive capacity, and strong hemostatic potential offer ideal characteristics to perform this surgery. We believe this technique can be reproduced using the T-1470 LiteTouch™ Laser in future patients.

Other fields that have employed a 1470 nm laser include otolaryngology, colorectal surgery, and vascular surgery [6–8]. In each of these fields, the laser was similarly selected for its strong hemostatic and ablative capacity. As the laser is relatively new, the nascent experience is growing, and literature regarding the use of this technology is limited.

**Figure 3:**
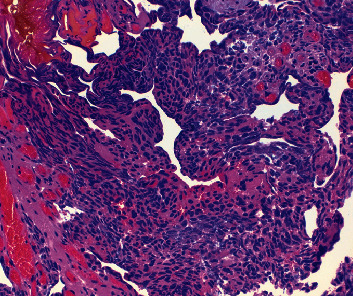
Marked thermal artifact; cell block (H&E, high power 40x).

While the aforementioned case illustrates a successful example of this procedure, there were several limitations encountered that should be noted to improve future procedures. Due to excess thermal injury, cautery artifact partially obstructed pathologic evaluation (Figure 3). This was likely a consequence of the frequency of the laser being set too high during this pilot experience. In the aforementioned case, the laser was set at 5 Watts and 30 Hz. The T-1470 LiteTouch™ Laser offers a 30 Hz, 20 Hz, and 10 Hz setting for frequency options. Reducing the frequency decreases the temperature during tissue en bloc resection and thereby reduces cautery artifact. One limitation of this approach is reduced tissue energy absorption at this frequency could potentially prolong procedural time. Nonetheless, for future procedures, we recommend decreasing the frequency to the 20 Hz or 10 Hz setting which will maintain the depth of penetration and hemostasis, as the wavelength is held constant, but minimize cautery artifact. In fact, the lower frequency increases the latency time between pulses and allows oxyhemoglobin more time to absorb the laser energy which theoretically may improve hemostasis.

Another limitation, to this novel approach, is identifying the appropriate margin of resection. Given en bloc resection of an upper tract urothelial carcinoma has not, to our knowledge, been performed previously, no literature exists to indicate an optimal margin. This limitation is further complicated by the grade, stage, architecture, use of neoadjuvant chemotherapy, and location of the tumor, all of which may affect the desired margin. One approach could be to resect a wide margin similar to that ablated during a laser ablation for low-risk disease. More data is needed to identify an appropriate margin.

## 4. Conclusion

Endoscopic management of UTUC offers a less invasive, kidney-sparing treatment for properly selected patients. The traditional endoscopic approach with Ho:YAG/Nd:YAG lasers is limited to just tumor ablation, given the limitations in tissue penetration depth and most importantly hemostasis. In this case, we illustrated the successful management of UTUC by tumor en bloc resection using the T-1470 LiteTouch™ Laser. Historically 1470 nm diode lasers have been used for tumor resection in other medical specialties such as otolaryngology [9]. Tumor en bloc resection using the T-1470 LiteTouch™ Laser is a novel technique and technology for the management of UTUC. The laser provided strong hemostasis and offered a potential advantage over current methods by yielding abundant tissue for accurate pathologic examination. As experience with this technology mounts, a more reliable assessment of the tumor grade and stage has the potential to expand the indication for endoscopic management to patients with higher risk features.

## References

[1] Petros F. G., Li R., Matin S. F. (2018). Endoscopic approaches to upper tract urothelial carcinoma. *The Urologic Clinics of North America*.

[2] Verges D. P., Lallas C. D., Hubosky S. G., Bagley D. H. (2017). Endoscopic treatment of upper tract urothelial carcinoma. *Current Urology Reports*.

[3] Breda A., Territo A., Sanguedolce F. (2019). Comparison of biopsy devices in upper tract urothelial carcinoma. *World Journal of Urology*.

[4] Rouprêt M., Babjuk M., Compérat E. (2018). European Association of urology guidelines on upper urinary tract urothelial carcinoma: 2017 update. *European Urology*.

[5] Shigemura F. (2018). Current status of holmium laser enucleation of the prostate. *International Journal of Urology*.

[6] Havel S. (2012). Intraindividual comparison of 1,470 nm diode laser versus carbon dioxide laser for tonsillotomy: a prospective, randomized, double blind, controlled feasibility trial. *Lasers in Surgery and Medicine*.

[7] Giamundo G. (2014). Closure of fistula-in-ano with laser - FiLaC™: an effective novel sphincter-saving procedure for complex disease. *Colorectal Disease*.

[8] Doganci S., Demirkilic U. (2010). Comparison of 980 nm laser and bare-tip fibre with 1470 nm laser and radial fibre in the treatment of great saphenous vein varicosities: a prospective randomised clinical trial. *European Journal of Vascular and Endovascular Surgery*.

[9] Liang F., Xiao Z., Chen R. (2019). Transoral 980-nm/1470-nm dual-wavelength fiber laser microsurgery for early-stage glottic carcinoma. *Oral Oncology*.

